# Rupture of an Aneurysmal Arteriovenous Fistula in an In Situ Vein Bypass

**DOI:** 10.3390/diagnostics12112566

**Published:** 2022-10-22

**Authors:** Hafedh Daly, Amira Horchani

**Affiliations:** 1 Cardiovascular Surgery Department, Regional Hospital (Gafsa), Faculty of Medecine, Monastir 5000, Tunisia; 2 Faculty of Pharmacy, Monastir 5000, Tunisia

**Keywords:** aneurysm, in situ saphenous vein, arteriovenous fistulas

## Abstract

We report a case of rupture of an aneurysm on a residual arteriovenous fistula, five years after a femoral to posterior tibial artery bypass for critical ischemia of the right lower limb. This is a 48-year-old patient admitted to the emergency room for hemorrhagic shock following a rupture of a varicose bundle at the level of the posterior surface of the right thigh. The CT angiography confirmed the presence of multiple residual arteriovenous fistulas, which became aneurysmal. The exclusion of the latter by ligature-section made it possible to stop the hemorrhage while preserving the bypass.

**Figure 1:**
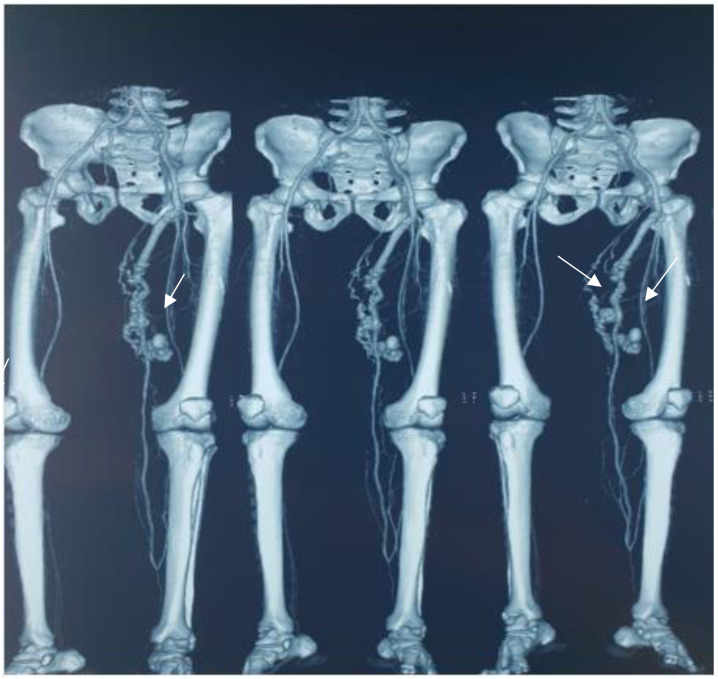
CT angiography of the lower limbs showing multiple arteriovenous fistulas complicating an in situ saphenous vein bypass. The femoral to posterior tibial artery bypass is a procedure used for revascularization of the lower limbs during critical limb ischemia. The conduit often used is the internal saphenous vein, which has a higher rate of patency than other vascular substitutes [1]. In situ saphenous vein bypass was first described by ROB [2]. The problem with this type of bypass is the persistence of a residual arteriovenous fistulas. These can cause localized edema, cellulitis, skin infarction, decreased distal flow and even graft thrombosis [3]. The rupture of an aneurysm of a residual arteriovenous fistula is an unusual late complication of an in situ saphenous graft motivated this report. A 48-year-old patient underwent a right femoral to posterior tibial artery bypass 5 years previously for critical lower limb ischemia. He was admitted to the emergency department for a ruptured varicose vein of the right lower limb with bright red blood. On examination, his blood pressure was 80/50 mm Hg, his heart rate was 110 cycles/minute and the presence of non-systematic varicose veins at the level of the lower third of the post face of the right thigh, surmounted by a small ulceration, was noted. In front of the antecedent of revascularization of the lower limb by a vein bypass, a CT angiography was done, which showed a patent bypass with multiple aneurysmal arteriovenous fistulas (Figure 1). The patient was operated on urgently. The surgical exploration confirmed the presence of multiple aneurysmal arteriovenous fistulas, which were excluded by section ligation (Figure 2). The postoperative course was simple.

**Figure 2:**
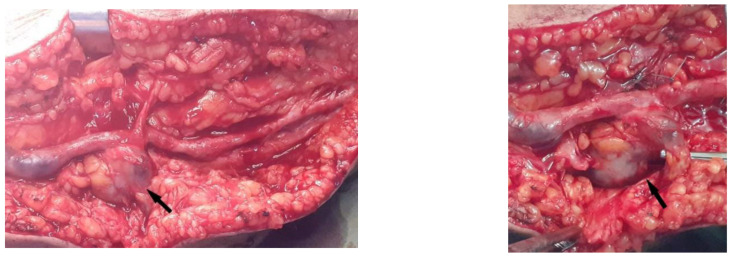
Intraoperative view showing the aneurysmal arteriovenous fistula. Aneurysmal degeneration of vein grafts is primarily pseudoaneurysms, with a small group consisting of true aneurysms [4]. The etiology of true aneurysms, which are rare, is atherosclerosis caused by smoking, hypertension, infections and hyperlipidemia [5]. In this report, we describe the unusual presentation of late autologous saphenous graft aneurysms related to a residual arteriovenous fistula after femoral to posterior tibial artery bypass surgery for critical ischemia of the lower limbs. The aneurysmal degeneration described here did not occur at an anastomotic site and therefore differs from common pseudoaneurysms. This case also differs from true venous conduit aneurysms that have been described due to atherosclerosis, intimal rupture, and post-stenotic dilatations. [6,7] Interestingly, the true vein graft aneurysms occur more frequently in the setting of popliteal artery aneurysmal diseases; however, there are very rare complications of bypass surgery for arterial occlusive disease [8]. Some authors have suggested that hemodynamic stress is an additional cause of true vein graft aneurysms [7,9]. The incidence of a residual arteriovenous fistula after an in situ bypass is 6–20% [10,11]. It has been implicated as a cause of bypass failure by some authors [12], although others have concluded that the persistent arteriovenous fistula does not affect the graft patency and that these connections thrombose spontaneously [11]. The femoral to posterior tibial artery bypass, in our case, remained patent despite the presence of these arteriovenous fistulas, which became aneurysmal. We suggest that the aneurysmal degeneration in this patient was likely the late results of hemodynamic changes due to the residual arteriovenous fistula. The rupture of an aneurysmal arteriovenous fistula from in situ saphenous vein bypass is extremely rare. No such case in the literature has been described. The seriousness of these complications is an additional argument for advocating regular clinical monitoring and echo-Doppler of patients who have had this type of bypass.

## Data Availability

The data that support the findings of this study are available from the corresponding author, H.D., upon reasonable request.
